# Long-Term Function, Pain and Medication Use Outcomes of Radiofrequency Ablation for Lumbar Facet Syndrome

**DOI:** 10.23937/2377-4630/2/2/1028

**Published:** 2015-04-06

**Authors:** Zachary L. McCormick, Benjamin Marshall, Jeremy Walker, Robert McCarthy, David R. Walega

**Affiliations:** 1 The Rehabilitation Institute of Chicago/Northwestern University Feinberg School of Medicine, Department of Physical Medicine and Rehabilitation, USA; 2 Northwestern University Feinberg School of Medicine, Department of Anesthesiology, USA

**Keywords:** Low back pain, Denervation, Zygapophyseal joint, Opioid analgesics

## Abstract

**Objective:**

Radiofrequency ablation (RFA) of the medial branch nerves for facet-mediated low back pain demonstrates clinical benefit for 6–12 months and possibly up to 2 years. This study investigated function, pain, and medication use outcomes of RFA for lumbar facet syndrome in a cohort with long-term follow-up.

**Methods:**

Individuals evaluated in a tertiary academic pain practice between January, 2007–December, 2013, 18–60 years of age, with a clinical and radiologic diagnosis of lumbar facet syndrome, who underwent ≥1set of diagnostic medial branch blocks with resultant >75% pain relief and subsequent RFA were included. Outcomes measured were the proportion of individuals who reported ≥50% improvement in function, ≥50% improvement in pain; change in median NRS pain score, daily morphine equivalent consumption (DME), Medication Quantification Scale III (MSQ III) score and procedure complications.

**Results:**

Sixty-two consecutive individuals with a median age and 25%–75% interquartile range (IQR) of 34 years (35, 52) met inclusion criteria. Seven individuals were lost to follow-up. Duration of pain was <2 years in 42%, 2–5 years in 40%, >5 years in 18% of individuals. Median duration of follow-up was 39 months (16, 60). Function and pain improved by ≥50% in 58% (CI 45%, 71%) and 53% (CI 40%, 66%) of individuals, respectively. The median reduction in MQS III score was 3.4 points (0, 8.8). No complications occurred in this cohort.

**Conclusions:**

This study demonstrates a durable treatment effect of RFA for lumbar facet syndrome at long-term follow-up, as measured by improvement in function, pain, and analgesic use.

## Introduction

Chronic low back pain is the leading cause of disability in the U.S. [1,2]. Lumbar zygapophyseal or “facet” joint pain has been estimated to account for as many as 30% of chronic low back pain cases [3]. Facet-mediated pain is typically related to osteoarthritis [4] with nociception originating in the synovial membrane, hyaline cartilage, bone, or fibrous capsule of the facet joint [5–7]. If facet-mediated pain is unresponsive to conservative management with oral non-steroidal anti-inflammatory drugs, physical therapy, and postural reeducation, interventional treatment may be indicated.

Nociceptive sensation in the facet joints is carried by afferent fibers in the medial branch nerves of the lumbar dorsal rami (MBN). Thus, lesioning of the MBN by radiofrequency ablation (RFA) is commonly used as a treatment for facet-mediated low back pain. RFA has been shown to provide significant improvement in function, pain, and analgesic use for 6–12 months in individuals with facet-mediated chronic low back pain [8–19]. Given the progressive nature of lumbar facet syndrome, and the lack of low risk, high value surgical options, defining the durability of treatment efficacy with RFA is important. However, most studies have investigated treatment outcomes at 1 year follow up or less and very few studies have reported outcomes beyond 2 year follow-up [20–23], with minimal assessment of changes in function [20,23] and analgesic use [21,23]. The purpose of this study was to investigate the outcome of RFA for the treatment of lumbar facet syndrome, as measured by function, pain, and medication usage at long-term follow-up.

## Methods

This was a prospective outcome study. The study protocol (STU00090028) was approved by the local Institutional Review Board and was conducted at a single-site interventional pain management practice in an urban tertiary academic medical center. Inclusion criteria were: age 18–60 years, low back pain from lumbar facet syndrome treated with RFA between January 1, 2007 and December 31^st^ 2013, history (axial low back pain), physical examination findings (no neurologic changes such as asymmetric lower extremity weakness or asymmetric muscle stretch reflexes, and no dural tensions signs), and imaging studies consistent with lumbar facet syndrome (facet arthropathy). Additionally, to be included, individuals had to have experienced >75% reduction in back pain symptoms following one set of diagnostic medial branch blocks (MBBs), or >75% pain reduction following a second set of confirmatory MBBs. A single set of positive MBBs (>75% pain relief), as opposed to dual comparative MBBs, has been established as a pragmatic clinical cut-off due to reduced cost [24,25], decreased risk of serious complications (epidural abscess, epidural hematoma, meningitis, etc) [5], and an acceptable false-positive rate in this context [26,27]. A second set of confirmatory MBBs was performed if patients experienced >50% relief, but <75% relief as has been previously recommended given the possibility of false negatives with 1 set of MBBs [28]. Patients with radicular symptoms, nerve root tension signs, lower extremity strength or reflex asymmetry were excluded from the study.

The medical records of these patients were reviewed and demographic data (age, sex, body mass index), duration of pain, radiologic diagnosis, and anatomic levels of RFA, pre-procedure pain scores and medication usage were recorded. These patients were then contacted by telephone and follow-up outcome data was obtained. Numerical Rating Scale (NRS) pain score, functional improvement, opioid and non-opioid medication use were collected with the use of a standardized questionnaire (Appendix A). If a patient could not be contacted by phone upon at least three attempts, on different days, at different times of the day, then the individual was considered “lost to follow up”. Our primary outcome measures were the rate of ≥50% functional improvement and the rate of ≥50% pain reduction at long-term follow up. Both function and pain were used as primary outcome measures as recommended by the National Institutes of Health [29].

### Procedures

Based on history, physical examination, and imaging studies, the treating physican selected the facet joints to be diagnostically blocked. The patient was blinded to the local anesthetic being used. A needle was placed at each target location (described below) and following confirmation of appropriate needle placement with fluoroscopy, 0.5 cc of 0.5% bupivacaine or 2% lidocaine was injected. The maximum number of diagnostic medial branch nerves blocked for any set of diagnostic injections was six. A postive response to a set of diagnostic medial branch blocks was defined as >75% reduction in back pain symptoms of concordant duration with the local anesthetic used.

**Figure 1:**
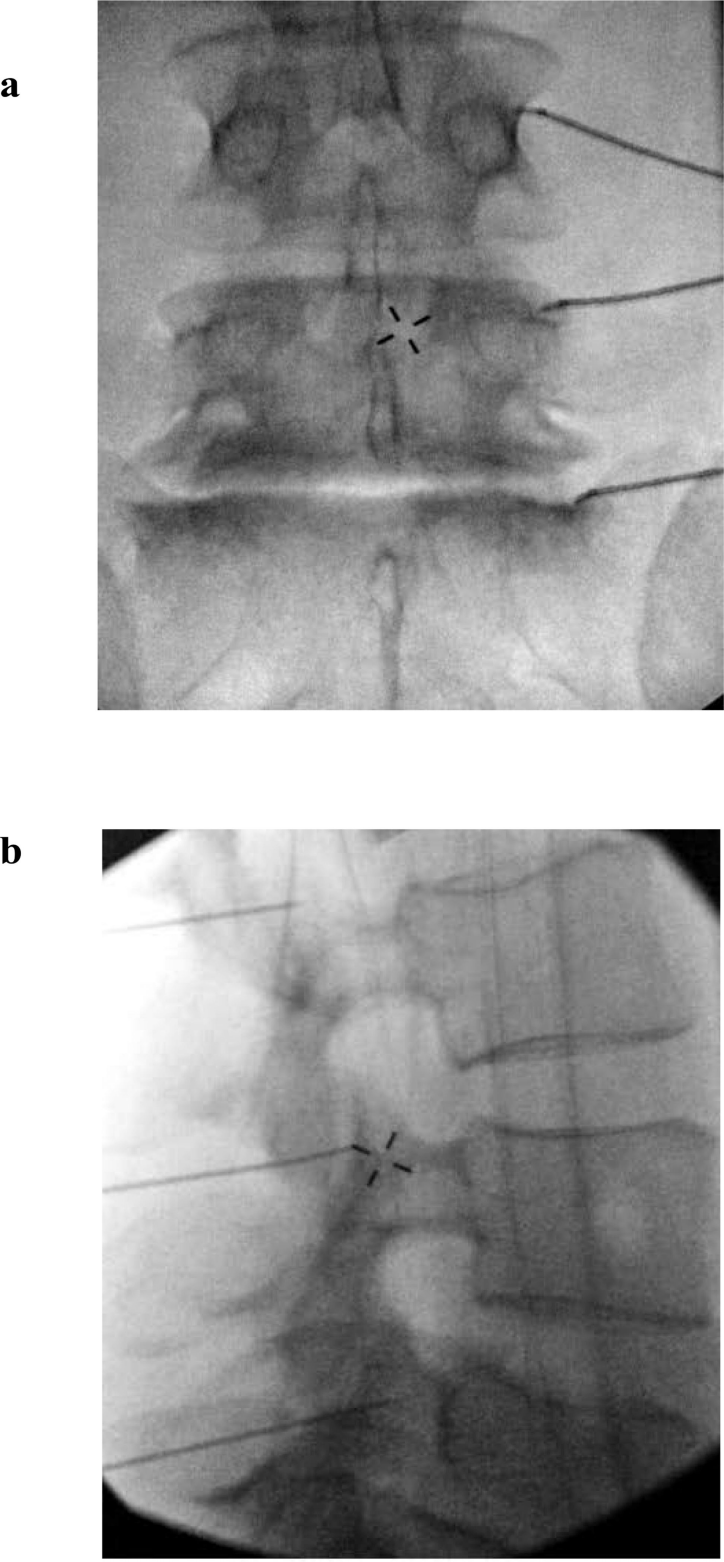
Anterior-posterior (1a) and lateral (1b) fluoroscopic views of the lumbar spine showing radiofrequency electrodes placed in parallel to the course of the L3 medial branch nerve, L4 medial branch nerve, and L5 dorsal ramus at their respective crossings of the L4 transverse process, L5 transverse process, and sacral ala.

At the time of the RFA procedure, patients were positioned prone on a fluoroscopy table and the lumbar region was prepped with chlorhexidine and draped in a standard sterile manner. Conscious sedation was used in some cases depending on physician or patient preference (midazolam 1–4mg IVP; fentanyl 50–100mcg IVP). After local anesthesia to the skin and subcutaneous tissues superficial to a planned target site, a 20 gauge 10cm RFA electrode with a 10mm active tip (Baylis Medical Company, Montreal Canada), was positioned using fluoroscopic guidance at the superior medial transverse process at the anatomic transition to the pedicle for the L1–L4 medial branches, and at the concavity of the sacral alae for the L5 medial branches. Care was taken to position the active tip of the electrode parallel to the expected course of the medial branch nerve as has been previously detailed in practice guidelines [30]. Correct electrode position was confirmed in both anterior-posterior and oblique fluoroscopic views following negative aspiration (Figure 1a,1b). Motor testing was performed at 2Hz to confirm the integrity of the corresponding exiting spinal nerve at each target. Sensory testing was performed at 50Hz to confirm proximity to the target MBN. After appropriate electrode positioning, 1cc of 2% lidocaine was injected through the introducer needle for anesthesia during the ablation. One RFA lesion was performed at each target site at 80°C for 90 seconds. Following the ablation, 0.5–1.0cc of 0.5% bupivacaine was injected to provide post-procedure analgesia. Following the procedure, patients were observed for approximately 30 minutes and were then discharged if clinically stable. Patients were asked to follow up in 4–6 weeks after the RFA procedure was performed.

### Data analysis

All collected data was entered into a password-protected database. Opioid medication doses for each patient were converted to daily morphine equivalents (ME) at each follow-up time point for comparisons. In addition, the Medication Quantification Scale (MQS) III, a validated equation used to objectively quantify medication use in pain management (including opioid and non-opioid medications) [31,32], was calculated for each patient at follow-up time points.

The number of individuals reporting ≥50% improvement in function, the number of individuals reporting ≥50% improvement in pain, the change in median daily ME, the change in MSQIII score, and the number of individuals who continued to seek treatment from other physician providers to treat low back pain were calculated and analyzed.

### Statistical analysis

Statistical software was used to analyze the data (SPSS, Version 22; Chicago, IL). Data were checked for distributional form and outliers using summary statistics and graphical displays. As data were not normally distributed, medians and 25%–75% interquartile ranges (IQR) were calculated and groups were compared with Mann-Whitney U tests for continuous variables. Proportions and 95% confidence intervals (CI) were calculated and groups were compared with Chi Square or Fisher Exact Tests for categorical variables. In order to determine effect size, median differences between groups were calculated using the 10,000 sample bootstrap method for continuous variables and percentage differences between groups were calculated using the Clopper-Pearson method. The level of significance was set at 0.05. Two-sided testing was used for all hypothesis testing.

## Results

Sixty-two consecutive individuals with a median age of 43 years (IQR 35, 52) were included in this study. Seven individuals were lost to follow-up. The duration of pain at the time of presentation was <2 years in 26 (42%), 2–5 years in 25 (40%), and >5 years in 11 (18%) individuals. The median baseline NRS pain score was 7 (IQR 5, 8). Demographic, clinical, and procedural characteristics of the study population are shown in Table 1.

The median duration of follow-up after RFA was 39 months (IQR 16, 60). Outcomes of RFA at this “long-term” time point are shown in Table 2. The proportion of patients who reported ≥50% improvement in function and pain were 58% (CI 45%, 71%), and 53% (CI 40%, 66%), respectively. Ten patients (18%) reported complete restoration of function and 19 (35%) reported at least 75% improvement in function. Five patients (9%) experienced complete pain reduction and 17 (31%) experienced at least 75% pain reduction. When using intention to treat analysis assuming treatment failure of all seven individuals who were lost to follow up, the proportion of patients who reported ≥50% improvement in function and pain were 52% (CI 40%, 64%) and 47% (CI 35%, 59%), respectively. The median reduction in MQS III score was 3.4 points (IQR 0, 8.8), a significant change from baseline (p=0.03).

A comparison of demographic, clinical, procedural, and outcome characteristics between patients who reported ≥50% versus <50% improvement in function at long-term follow-up is shown in Table 3. Individuals who underwent a repeat RFA, experienced ≥50% improvement in pain, or reported larger median decreases in NRS pain scores were significantly more likely to report ≥50% improvement in function (p<0.01; p<0.01; p<0.01, respectively). Women were significantly more likely to experience ≥50% improvement in function (p=0.01).

Due to the sex difference observed in functional improvement, a comparison of men versus women who reported ≥50% versus <50% improvement in function at long-term follow-up was also performed. There was no difference between the proportion of males and females in each group with respect to those who experienced ≥50% pain reduction and those who did not (p=0.88).

Individuals who reported significant functional improvement (≥50% “functional responders”) were 32% (CI 2%–62%), more likely to also experience significant pain reduction (≥50%) and reported a median 2-point (CI 1, 5), greater decrease in low back pain on the NRS compared to individuals who were not functional responders. Functional responders were also 36% (CI 9%–63%) more likely to have undergone a repeat RFA procedure for their low back pain, compared to non-responders.

Sub-analysis of functional, pain, and analgesic use outcomes in individuals who underwent one versus two sets of diagnostic MBBs was performed (Table 4). Seventeen percent and 16% more individuals who underwent two sets of diagnostic MBBs experienced ≥50% improvement in function and ≥50% improvement in pain at long-term follow-up, respectively; however, these differences were not statistically significant (p=0.22, p=0.21 respectively).

There was no significant interaction between repeat RFA procedures and pain reduction at 6 week or 39 month follow-up (p=1.0; p=0.94 respectively). This was true when analyzing both categorical (proportion of individuals with ≥50% reduction in pain) and continuous (change in median NRS pain score) data. No adverse events related to the RFA procedure occurred in this cohort.

## Discussion

While RFA of the medial branch nerves has been shown to improve function, pain, and analgesic use for 6–12 months in patients with lumbar facet syndrome [8–19], to our knowledge, the present study is the first to assess this battery of outcomes at greater than 3 year follow-up. These data demonstrated clinically significant improvements in self-reported function, pain, and analgesic use at a median follow-up over 3 years.

The studies with the longest duration of follow-up [20–23], prior to the present investigation, defined meaningful pain relief categorically as either ≥50% [20–22] or >80% [23] pain reduction. Two of these studies demonstrated a 45–55% proportion of patients with meaningful pain reduction (≥50%) at average or median 2-year follow-up (North, Park). One study found a 56% proportion of patients that experienced meaningful pain relief (>80%) at a median follow-up of 33 months [23]. The data in the present study indicate minimal degradation of pain relief with a 53% proportion of individuals reporting ≥50% pain reduction at median follow-up exceeding 3 years by standard analysis and a 47% proportion by intention-to-treat analysis.

There was a greater likelihood of long-term improvement in function and pain if the RFA procedure was repeated (Table 3). It is known that re-inervation of the facet joint from neural re-growth occurs after medial branch nerve RFA, a process with duration proportional to the size of the thermal lesion [33]. Each additional RFA treatment is associated with approximately 10–16 months of improvement in symptoms in patients who received benefit from the first procedure [23,34–36]. The present study provides support to the feasibility of using appropriately repeated RFA for long-term treatment of lumbar facet syndrome.

Individuals who underwent two rather than only one set of diagnostic MBBs were 17% and 16% more likely to experience improvement in function and pain, respectively. While these differences were not statistically significant, this study was not powered for this sub-analysis, and we suspect that studying a larger sample of patients would demonstrate statistical significance. This study suggests that dual compared to single diagnostic MBBs may result in improved outcomes of RFA at long-term follow-up, however, further comprehensive research is needed as this remains a controversial clinical decision. Although dual comparative MBBs with responses of >75–80% have been recommended [33,37,38], others report that one set of blocks is sufficient to proceed with RFA [26,27], particularly in the context of reduced cost [24,25] and complication rates [5]. Additionally, some insurers will not pay for a second set of confirmatory MBBs, thus while dual MBBs decrease the chance of false positive diagnoses of lumbar facet syndrome, in a realistic busy clinical practice, the dual block paradigm may not be practical.

Although many assume a strong relationship between function and pain, the two only correlate weakly in many patients with chronic low back pain [39,40]. In this study, improvement in function was strongly associated with reduction in pain (Table 3). However, this was not the case with regard to sex differences. A significantly larger proportion of women reported ≥50% functional improvement compared to men, where as there was no difference with regard to categorical pain reduction. This may reflect our use of a self-reported subjective measure of function. While sex differences in the perception of pain are well described, to the best of our knowledge, there is no literature that addresses sex differences in the correlation between pain and function. Further research in this area is needed, as this impacts clinical outcome assessment of interventions that are meant to improve both pain and function.

These data also demonstrate a reduction in MSQ III score equivalent to a patient discontinuing 1800mg of ibuprofen daily or 10mg of hydrocodone daily. Changes in analgesic use ranges 0–80% in the RFA literature [4,9,10,21] and is likely related to variable follow-up intervals and use of non-validated measures to assess analgesic use [32]. In the present study, DME was reduced from baseline to long-term follow-up, but the difference did not reach statistical significance. This may be related to a low baseline consumption of 10 DME, which leaves little room for improvement. Prior authors have described this as the “healthy person effect” [41]. Additionally, opioid prescribing habits are highly correlated with physician preference or other immeasurable patient or cultural factors. Further study of the effects of RFA on opioid consumption is needed, particularly in patients who have been taking opioid medications chronically.

An important strength of the current study is the assessment of function as a primary clinical outcome. Assessment of function in studies of chronic low back pain is vital given that this condition is the leading causes of disability and work absenteeism in the U. S. [1,2]. The finding of functional improvement in a large proportion of this cohort (58%) is particularly notable given the mixed results and shorter duration of follow-up of functional outcome measurement in prior studies [9–11,14,16–18].

## Study Limitations

Classification bias is a possible limitation of this study with the use of a conditional one versus two diagnostic MBB protocol. The 51% of patients who received only 1 set of MBBs are at risk of false positive diagnosis, and thus, thus this study may underestimate the effectiveness of RFA with a dual diagnostic MBBs screening paradigm. However, as discussed above, the use of single versus dual diagnostic MBB is controversial. Thus, this study provides insight into the expected clinical outcomes of RFA treatment when using a pragmatic screening protocol.

Additionally, we used self-reported percentage-based improvement in function as a primary outcome, but using specific and sensitive validated measures of function would strengthen future investigation. We did not assess the cost-effectiveness or impact on healthcare utilization. Few studies in the RFA literature have addressed these outcomes. In a study which analyzed the costs of pain care following RFA for lumbar facet syndrome as a secondary outcome measure, costs (the sum of physician office visits, chiropractic treatments, physical therapy treatments, and treatments from other allied health practitioners) were decreased for up to nine months following the procedure compared to the sum costs of care during a time-period of equal duration prior to the procedure [13]. Future studies should evaluate both the direct and indirect costs and potential cost-effectiveness of this procedure.

## Conclusions

We report the first study of RFA for the treatment of lumbar facet syndrome at long-term follow-up. We found significant improvements in self reported function, pain, and analgesic use following this procedure.

## Figures and Tables

**Table 1:** Baseline demographic, clinical, and procedural information (n=62).

	Median (IQR) or n (%)

Age (years)	43 (35, 52)

Sex	
Male	33 (53%)
Female	29 (47%)

BMI (Kg/m^2^)	27 (23, 30)

Duration of pain at presentation	
<2 years	26 (42%)
2–5 years	25 (40%)
>5 years	11 (18%)

NRS pain score	7 (5, 8)

Morphine Eq	10 (0, 15)

MQS III score	10.6 (4.6, 14.8)

Number of diagnostic MBB blocks	
1	29 (47%)
2	33 (53%)

Number of facet joint levels denervated	
1	15 (24%)
2	28 (45%)
3	19 (31%)

Bilateral procedures	36 (58%)

RFA procedure repeated	
Yes	27 (44%)
No	35 (56%)

BMI: Body Mass Index, Eq: Equivalents, IQR: Interquartile Range, MBB: Medial Branch Block, MQS III score: Medication Quantification Scale III score, NRS: Numerical Rating Scale, RFA: Radiofrequency Ablation

**Table 2:** Long-term outcomes of radiofrequency ablation procedure (n=55).

	Median (IQR) or Percent [95% CI]
Duration between procedure and follow up (months)	39 (16,60)
≥50% patient perceived functional improvement	58% [45%,71%]
≥50% reduction in pain	53% [40%,66%]
Reduction in NRS pain score	2 (1,5)
Reduction in morphine eq	0 (0, 10)
Reduction in MQS III score	3.4 (0,8.8)

CI: Confidence Interval, Eq: Eequivalents, IQR: Interquartile Range, MQS III score: Medication Quantification scale III score, NRS: Numerical Rating Scale

**Table 3:** Demographic, clinical, procedural and outcome characteristics in patients who experienced ≥50% functional improvement compared to patients with <50% functional improvement at long-term follow up (n=55 total patients).

	≥50% patient perceived functional improvement (n=32) Median (IQR) or n (%)	<50% patient perceived functional improvement (n=23) Median (IQR) or n (%)	P value

Age (years)	43 (33, 53)	44 (39, 53)	0.43

Sex			
Male	10 (31%)	16 (70%)	
Female	22 (69%)	7 (30%)	**0.01**

BMI (Kg/m^2^)	25 (22, 30)	27 (23, 30)	0.63

Duration of pain at presentation			
<2 years	14 (44%)	11 (48%)	
2–5 years	10 (31%)	8 (35%)	
>5 years	8 (25%)	4 (17%)	0.79

Number of diagnostic MBB blocks			
1	14 (44%)	13 (57%)	
2	18 (56%)	10 (43%)	0.42

Number of facet joint levels denervated			
1	6 (19%)	10 (43%)	
2	15 (47%)	10 (43%)	
3	11 (34%)	3 (14%)	0.07

Bilateral procedures	14 (44%)	11 (48%)	0.38

Repeat RFA	13 (46%)	2 (9%)	**<0.01**

Duration between procedure and follow up (months)	36 (16, 63)	39 (16, 54)	0.47

Baseline NRS pain score	7 (5, 8)	6 (5, 8)	0.47

Reduction in NRS pain score	3 (1, 7)	1 (0, 2)	**<0.01**

≥50% reduction in pain	26 (87%)	4 (17%)	**<0.01**

Baseline Morphine eq	10 (0, 10)	10 (0, 26)	0.11

Reduction in Morphine eq	2.5 (0, 10)	0.0 (0, 5)	0.72

Baseline MQS III score	11.2 (6.8, 16.4)	10.6 (5.6, 14.8)	0.78

Reduction in MQS III score	4.8 (0, 11.1)	0.0 (−7.6, 4.5)	0.17

BMI: Body Mass Index, CI: Confidence Interval, Eq: Equivalents, IQR: Interquartile Range, MBB: Medial Branch Block, MQS III score: Medication Quantification scale III Score, NRS: Numerical Rating Scale, RFA: Radio Frequency Ablation

**Table 4:** Outcome in patients who had 1 versus 2 sets of diagnostic medial branch blocks prior to radiofrequency ablation (n=55 total patients).

	1 Set of Medial Branch Blocks (n=28) Median (IQR) or n (%)	2 Sets of Medial Branch Blocks (n=27) Median (IQR) or n (%)	P value
≥50% improvement in function	13 (46%)	17 (63%)	0.22
Reduction in NRS pain score	2 (1, 5)	2 (0, 4)	0.56
≥50% improvement in pain	12 (43%)	16 (59%)	0.21
Change in Morphine eq	0 (0, 10)	1.7 (0, 10)	0.57
Change in MQS III score	2.7(0, 9.6)	3.4 (0, 11.6)	0.35

Eq: Equivalents, IQR: Interquartile Range, MQS III score: Medication Quantification Scale III score NRS: Numerical Rating Scale

## Appendix A

1. Are you currently having low back pain (pain complaint from medical record) pain today?
2. How would you score your average pain over the past week on a scale of zero to 10, zero being no pain at all, and 10 being the absolute worst pain imaginable?
3. Is this the same pain you were treated for at the Pain Center?
4. Can you quantify the amount of improvement in pain as a percentage? For example, is your pain 10% improved? 50% improved? 75% improved?
5. Has your physical function, like activities of daily living, exercise and leisure, or ability to physically function at work improved since the RF nerve ablation procedure at the Pain Center?
6. Can you quantify the amount of functional improvement as a percentage? For example, is your function 10% improved? 50% improved? 75% improved?
7. What medications do you take for this pain now? (include analgesics, NSAIDs, SNRI, TCA, topicals) What are the doses and how many do you use daily?
